# Prognostic impact of FAS/CD95/APO-1 in urothelial cancers: decreased expression of Fas is associated with disease progression

**DOI:** 10.1038/sj.bjc.6602732

**Published:** 2005-08-09

**Authors:** K Yamana, V Bilim, N Hara, T Kasahara, T Itoi, R Maruyama, T Nishiyama, K Takahashi, Y Tomita

**Affiliations:** 1 Division of Molecular Oncology, Department of Signal Transduction Research, Niigata University Graduate School of Medical and Dental Sciences, Asahimachi 1-757, Niigata 951-8510, Japan; 2 Division of Urology, Department of Metabolic and Regenerative Medicine, Yamagata University School of Medicine, Iida-nishi 2-2-2, Yamagata 990-9585, Japan; 3 Division of Urology, Department of Regenerative and Transplant Medicine, Niigata University Graduate School of Medical and Dental Sciences, Asahimachi 1-757, Niigata 951-8510, Japan

**Keywords:** Fas, Fas ligand, Decoy receptor 3, urothelial cancer, anti-Fas monoclonal antibody, adriamycin

## Abstract

The death receptor Fas (Apo1/CD95) and Fas ligand (FasL) system is recognised as a major pathway for the induction of apoptosis *in vivo*, and antiapoptosis via its blockade plays a critical role in carcinogenesis and progression in several malignancies. However, the function of Fas–FasL system in urothelial cancer (UC) has not been elucidated. We therefore investigated the expression of Fas, FasL and Decoy receptor 3 for FasL (DcR3) in UC specimens and cell lines, and examined the cytotoxic effect of an anti-Fas-activating monoclonal antibody (mAb) *in vitro*. Immunohistochemical examinations of Fas-related molecules were performed on 123 UC and 30 normal urothelium surgical specimens. Normal urothelium showed Fas staining in the cell membrane and cytoplasm. In UC, less frequent Fas expression was significantly associated with a higher pathological grade (*P*<0.0001), a more advanced stage (*P*=0.023) and poorer prognosis (*P*=0.010). Fas and the absence thereof were suggested to be crucial factors with which to select patients requiring more aggressive treatment. Moreover, low-dose anti-Fas-activating mAb sensitised resistant cells to adriamycin, and this synergistic effect could be applied in the development of new treatment strategy for UC patients with multidrug-resistant tumours.

Urothelial cancer (UC), which arises from the urothelium all the way from the renal calices to urethra, is the second most frequent urological malignancy after prostate cancer. Transitional cell cancer (TCC), which is the most frequent type of UC, accounts for more than 90% of all bladder cancers (Epstein *et al*, 1998). Approximately 70% of tumours are superficial at initial diagnosis, but of those about 60% recur and 10–20% of these progress (Ro *et al*, 1992). Whereas survival is excellent in patients with superficial disease, it falls substantially accordingly as the tumour progresses. As representative prognostic markers, pathological grade and tumour stage are very useful. However, as grade 2 UC are still heterogeneous, there is a necessity to find new markers to subclassify grade 2 tumours. In the treatment of advanced stage bladder cancers, combination chemotherapy including platinum has revealed promising results (Mead *et al*, 1998). However, multidrug-resistant bladder cancer shows a poor prognosis. Therefore, attempts to establish new treatment modalities are being made. Bacille Calmette-Guerin (BCG) intravesical instillation is an effective therapy for superficial UC, suggesting that utilisation of the immune system may contribute to new treatment strategies.

Two major intracellular apoptotic signalling pathways are known. In the type 1 cascade, death ligand–death receptor ligation activates downstream death signals through caspase-8. The type 2 cascade is a mitochondrial pathway mainly regulated by the Bcl-2 family proteins. Fas (Apo1/CD95), TNFR-1, DR3, DR4 and DR5 are well known as death receptors, and ligation by their specific ligands delivers a direct and powerful signal that rapidly induces apoptosis. Fas ligand (FasL) is produced by activated T cells and natural killer (NK) cells, and it triggers the downstream apoptotic signals through Fas. The Fas–FasL system is recognised as a major pathway for the induction of apoptosis in cells and tissues (Nagata, 1997). Fas is widely expressed in normal and neoplastic cells, but the expression of this protein does not necessarily predict susceptibility to apoptosis. Recent studies suggested that resistance to apoptosis with the Fas blockade might play an important role in tumorigenesis and tumour progression in several malignancies, including colorectal cancer (O'Connel *et al*, 1996) and malignant glioma (Roth *et al*, 2001).

Several mechanisms for evading the Fas–FasL system have been proposed. Principally, FasL-bearing tumour cells can induce Fas-mediated apoptosis of antitumour lymphocytes bearing Fas, which is known as the Fas–FasL tumour counterattack theory (O'Connel *et al*, 1996). The validity of the Fas–FasL counterattack theory has been studied in several malignancies, and FasL expression could be a negative prognostic factor, such as in colorectal cancer (Mann *et al*, 1999; Okada *et al*, 2000). Second, decoy receptor 3 (DcR3) (TR6, M68), a decoy receptor for FasL, can play an important role in various cancers (Pitti *et al*, 1998; Roth *et al*, 2001). Moreover, recent study demonstrated that DcR3 expression might be a clinical marker in colorectal cancer (Mild *et al*, 2002). The caspase-8 homologue FLICE-inhibitory protein (FLIP) functions as a caspase-8 dominant negative regulator, blocking signal transduction downstream of Fas (Irmler *et al*, 1997).

In the current study, we investigated the expression of Fas, FasL and its decoy receptor DcR3 in 30 normal urothelium specimens, 123 UC surgical specimens and four UC cell lines of different malignant potential. Moreover, we examined the association of Fas expression with the cytotoxic effect of anti-Fas monoclonal antibody (mAb) and adriamycin (ADM) *in vitro*. Among these three Fas-related molecules, loss of Fas may have the greatest impact on tumour progression leading to a poorer prognosis among UC patients.

## PATIENTS AND METHODS

### Patients

A total of 123 patients with UC including bladder cancer (86), ureteral cancer (20) and renal pelvic cancer (18), who underwent surgical resection at our institution from 1987 to 2002, were subjected to this study. Informed consent to perform the study was obtained from all patients. Patients' characteristics are presented in Table 1. We selected only sequential cases of TCC, in which tumour specimens were available, in this study. Histopathological grading and staging were performed according to the 1999 WHO and 1997 TNM classification, respectively.

### Cells

Four established UC cell lines were obtained from the American Type Culture Collection (ATCC). T24 (TCC), HT1376 (TCC) and SCaBER (squamous cell carcinoma) were derived from high-grade bladder cancer; the other, RT4 (TCC), was derived from low-grade cancer. T24 is known to be multidrug resistant. DNA, RNA and protein were extracted from cells when they reached subconfluence according to protocols described previously (Tomita *et al*, 2003). We used Jurkat, a lymphoma cell line, and SW480, a colon cancer cell line, as controls. The former is well known as Fas expressing cell, and the latter expresses various death receptors.

### Reagents and antibodies

The four anti-Fas monoclonal antibodies (mAb) were used. Apoptosis-inducing anti-Fas IgM (clone CH-11) and Fas-blocking IgG (clone ZB4) from the Medical Bioscience Laboratory (MBL), Nagoya, Japan were used for *in vitro* assays and flow cytometry. Anti-Fas clone UB2 (MBL) and clone 13 (BD Trasduction Laboratories, Tokyo, Japan) were used for immunohistochemistry. There was no difference between these two antibodies in immunoreactivity. Anti-FasL IgG clone Q-20 (Santa Cruz Biotechnology, Santa Cruz, CA, USA), anti-DcR3 IgG (Upstate biotechnology, Lake Placid, NY, USA), anti-caspase-8 IgG (MBL), anti-FLIP IgG (ALEXIS, Tokyo, Japan) and anti-Bid Ig (BD, Tokyo, Japan) were used for immunoblotting and immunohistochemistry. Biotinylated secondary antibody, sheep anti-mouse and streptavidin-horse radish peroxidase (HRP) were purchased from Amersham, Aylesburg, UK, and HRP-conjugated goat anti-rabbit was from Dako, Glostrup, Denmark. Adriamycin (Doxorubicin hydrochloride) was purchased from Kyowa Hakko Kogyo, Tokyo, Japan.

### Reverse transcription–polymerase chain reaction (RT–PCR)

Reverse transcription–polymerase chain reaction was performed according to a previously described protocol, with modifications (Hara *et al*, 2002). Total RNA was extracted using ISOGEN (Nippongene, Tokyo, Japan), according to the manufacturer's instructions. In total, 2 *μ*g of total RNA was used for the synthesis of cDNA with a first-strand cDNA synthesis kit (Roche Diganostics, Tokyo, Japan). The first strand cDNA (1 *μ*l) was then used for PCR with primers designed to amplify the target sequences given in Table 2. Amplification was carried out with an initial denaturation at 94°C for 5 min, followed by 35 cycles of 1 min denaturation at 94°C, 1 min annealing at the temperature listed in Table 2, and 1 min extension at 72°C, then a final extension for 5 min at 72°C.

### Real-time quantitive PCR

High molecular weight DNA was isolated from tumour tissues and cell lines using the phenol–chloroform extraction method. Real-time PCR detection was performed in triplicate on the ABI Prism 7700 using a TaqMan instrument (Applied Biosystems, Tokyo, Japan) as previously described (Tomita *et al*, 2003). DcR3 nucleotide sequences for primers are described previously (Pitti *et al*, 1998). To enable the standardisation of DNA quantity, TaqMan *β*-actin real-time PCR was performed for each sample, and the results were used to adjust the final results of real-time PCR for DcR3. Each normalised target value was divided by the calibrator-normalised target value to calculate the relative expression level.

### Immunoblotting and immunohistochemistry

Immunoblot analysis was performed as described previously (Tomita *et al*, 2003). The cell lysates were subjected to electrophores on 12 or 15% SDS–polyacrylamide gels, then electroblotted to Hybond-ECL super membranes (Amersham). The blots were blocked with 5% nonfat milk, and then incubated with the antibodies described above. After three washes with Tris-buffered saline (pH 7.6), containing 0.1% Tween-20, the blotted membranes were incubated with biotinylated secondary antibodies and subsequently with peroxidase-conjugated streptavidin (Amersham). Visualisation of stained blots was performed with the ECL Western Blotting Detection system (Amersham) according to the manufacturer's instructions. Immunohistochemical staining was performed using the avidin–biotin peroxidase complex method with 5 *μ*m cryostat sections from frozen tissue as described earlier (Itoi *et al*, 2004). We classified the immunoreactivity as positive or negative. We observed two different patterns of staining: a homogeneous strong staining or heterogeneous staining of more than 60% of all cells, which we defined as positive; and less than 60% positive staining in tumour cells, which was defined as a negative pattern. There were no intermediate pattern samples and most of negative tumours showed less than 25% positive cells. Infiltrating lymphocytes showing Fas expression were used as an internal positive control.

### *In vitro* cell toxic assay

We examined the possible modulation of Fas and FasL expression following ADM treatment in RT4 and T24. The reasons we selected these cell lines are that both of them are derived from transitional cells and similar in Fas, FasL and DcR3 status, while the former is susceptible to ADM and the latter is highly resistant. The MTT conversion assay (Chemicon International Inc., Temecula, CA, USA) was used to estimate cell viability (Tomita *et al*, 2003). After trypsinisation and washing, cells were resuspended in the medium, and added to a 96-well flat-bottom plate (167008 Nunk, Roskilde, Denmark), at 5 × 10^3^ cells per well in 100 *μ*l of the medium. The cells were cultivated at 37°C with 5% CO_2_ for 3 h and then subjected to treatment with anti-Fas antibodies in combination with ADM or without it. At 4 h prior to the end of the assay, 10 *μ*l of MTT solution was added to each well, containing both attached viable cells and detached apoptotic cells, and the incubation was carried out for 4 h. All functional experiments were accompanied with a control – Jurkat cells. Absorbance was evaluated at 570 nm with a Multiplate Reader (Bio Rad, Hercules, CA, USA). Each dose was applied in six wells and the mean and standard deviation were calculated. Each experiment was repeated at least three times.

### Flow-cytometric analysis

For flow-cytometric analysis, cells were stained by the indirect immunofluorescence method as described (Bilim *et al*, 2003). Briefly, tumour-cell suspensions were prepared by treatment with 0.125% trypsin and 0.02% EDTA. Tumour cells (1 × 10^6^) were reacted for 30 min at 4°C with anti-Fas mAb in PBS supplemented with 2% FCS and 0.02% sodium azide. After two washes by centrifugation, cells were incubated with FITC-conjugated rabbit anti-mouse Ig (DAKO) for 30 min at 4°C. Subsequently, the cells were washed three times and suspended at a concentration of 1 × 10^6^ cells ml^−1^ and analysed by flow cytometry (FACScanTM, Becton Dickinson, Mountain View, CA, USA). Jurkat cells, known to express Fas on the cell membrane, were used as a positive control. As negative control, first antibody was substituted with mouse Ig (DAKO).

### Statistical analysis

Statistical analysis was performed using Stat View 5.0 for Macintosh. Associations of Fas, FasL and DcR3 expression with clinicopathologic parameters were tested with chi-square test and Fisher's exact test of variance. The survival analysis was performed using Kaplan–Meier method as the end point for disease-specific survival, and the differences between groups were tested using log-rank test.

## RESULTS

### Expression of Fas, FasL, DcR3 and other death receptors in human UC cell lines

**Figure 1:**
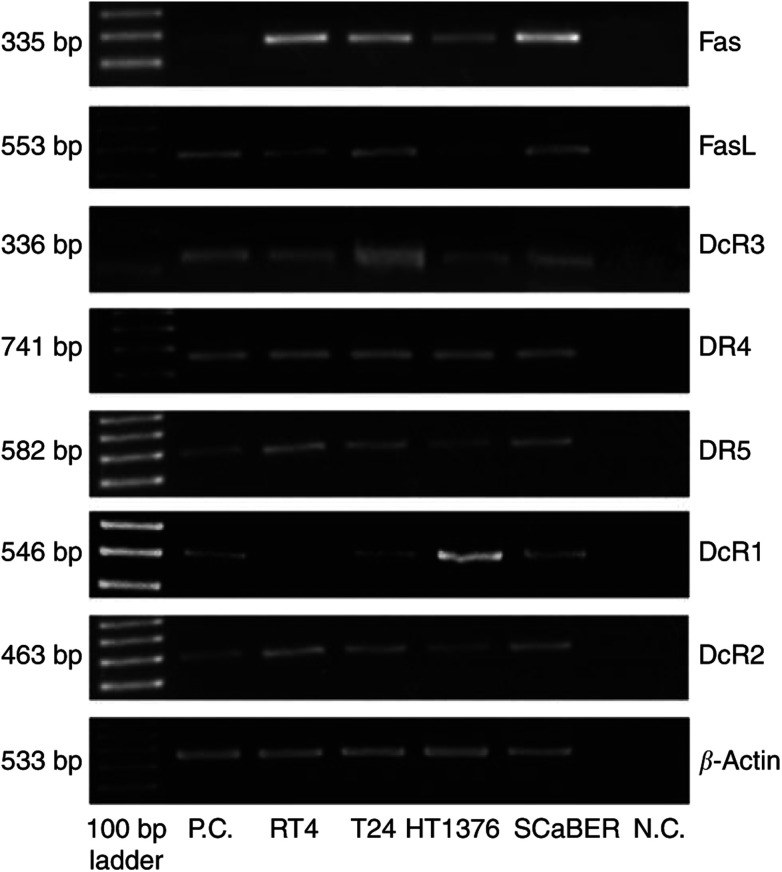
Reverse transcription–PCR of Fas-related molecules, Fas, FasL and DcR3 and other death receptors in human bladder cancer cell lines. The primer sequences and annealing temperatures are listed in Table 2. We selected Jurkat (Fas, DcR2) and SW480 (FasL, DcR3, DR4, DR5, DcR1) as positive controls (P.C.). Negative control (N.C.) without cDNA template was also applied.

**Figure 2:**
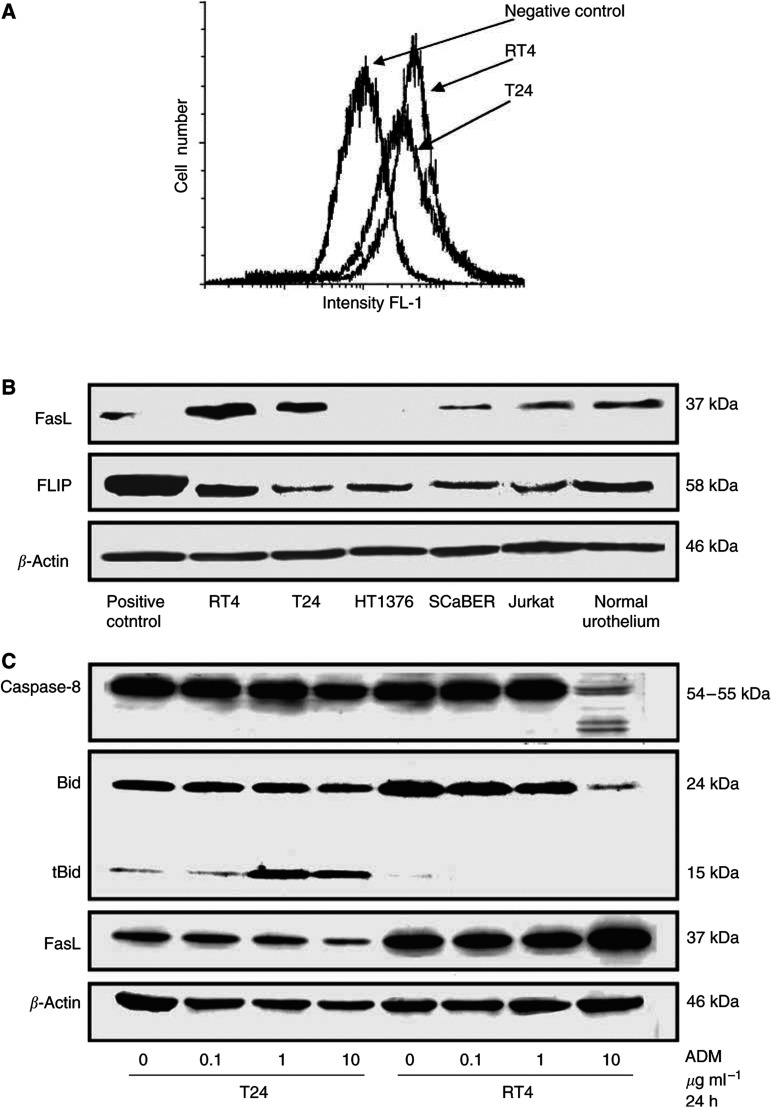
(**A**) FACScan analysis for cell surface Fas in T24 and RT4 cell lines. (**B**) Immunoblotting for FasL and FLIP in UC cell lines and normal urothelium compared with two control cell lines; SW480 to FasL and HEK 293T cells transfected with human FLIP. A single band of 37 kDa for FasL and 58 kDa for FLIP were detected. *β*-Actin was applied as a control for loading. (**C**) Immunoblotting for FasL, caspase-8 and Bid in T24 and RT4 cell lines following ADM treatment.

The expression of Fas, FasL and DcR3 was assessed by RT–PCR (Figure 1). The expression of other death receptors, DR4 and DR5, and decoy receptors, DcR1 and DcR2, was assessed by RT–PCR. All cell lines expressed these molecules at different levels (Figure 1). All four cell lines expressed equal levels of Fas, FasL and DcR3 except HT1376 cells which showed much less FasL. Expression of cell surface Fas was confirmed at the protein level by FACS analysis, and the levels were the same among the examined cell lines (Figure 2A). FasL expression was confirmed at the protein level by immunoblotting, and the expression levels were variable (Figure 2B).

### Anti-Fas mAb and ADM treatment in UC cell lines

**Figure 3:**
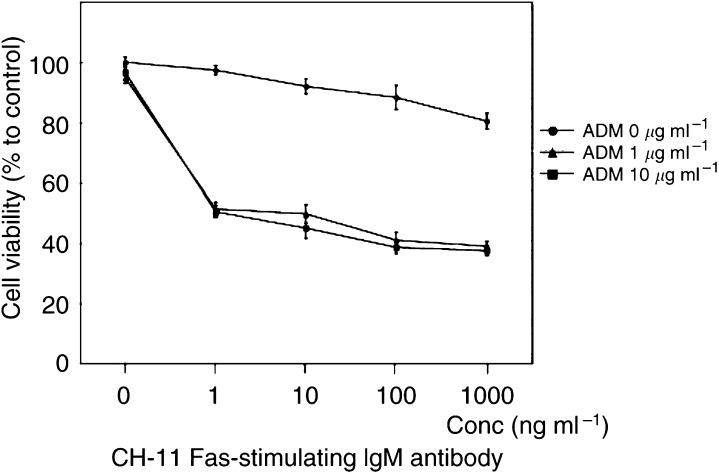
MTT cell viability assay of T24 cells treated with either ADM or CH-11 Fas-activating antibody, or a combination of both. Adriamycin or/and CH-11 were added to the culture medium at the indicated concentrations and MTT assay was performed 24 h later.

We examined the possible modulation of Fas and FasL expression following ADM treatment in RT4 and T24. We did not find any upregulation of Fas expression after the exposure to ADM (data not shown). On the other hand, the exposure resulted in a downregulation of FasL expression in T24 but not RT4. In addition, proapoptotic member of Bcl-2 family proteins, Bid was processed to its active form tBid in a dose-dependent manner in T24, although cell death was not induced. ADM triggered procaspase-8 cleavage into active subunits in RT4 (Figure 2C). ZB4 Fas-blocking antibody did not affect the viability of either naïve or ADM-treated cells (data not shown). Treatment with CH-11 Fas-activating antibody did not have any effect on the viability of T24 cells except at an extremely high concentration (1000 *μ*g ml^−1^) at which viability decreased by less 20% (Figure 3). Double treatment with CH-11 and ADM resulted in a synergistic effect in both T24 and RT4. This synergistic effect started from 1 ng ml^−1^ of CH-11 and 1 *μ*g ml^−1^ of ADM (Figure 3).

### Fas expression in normal urothelia and UC specimens

**Figure 4:**
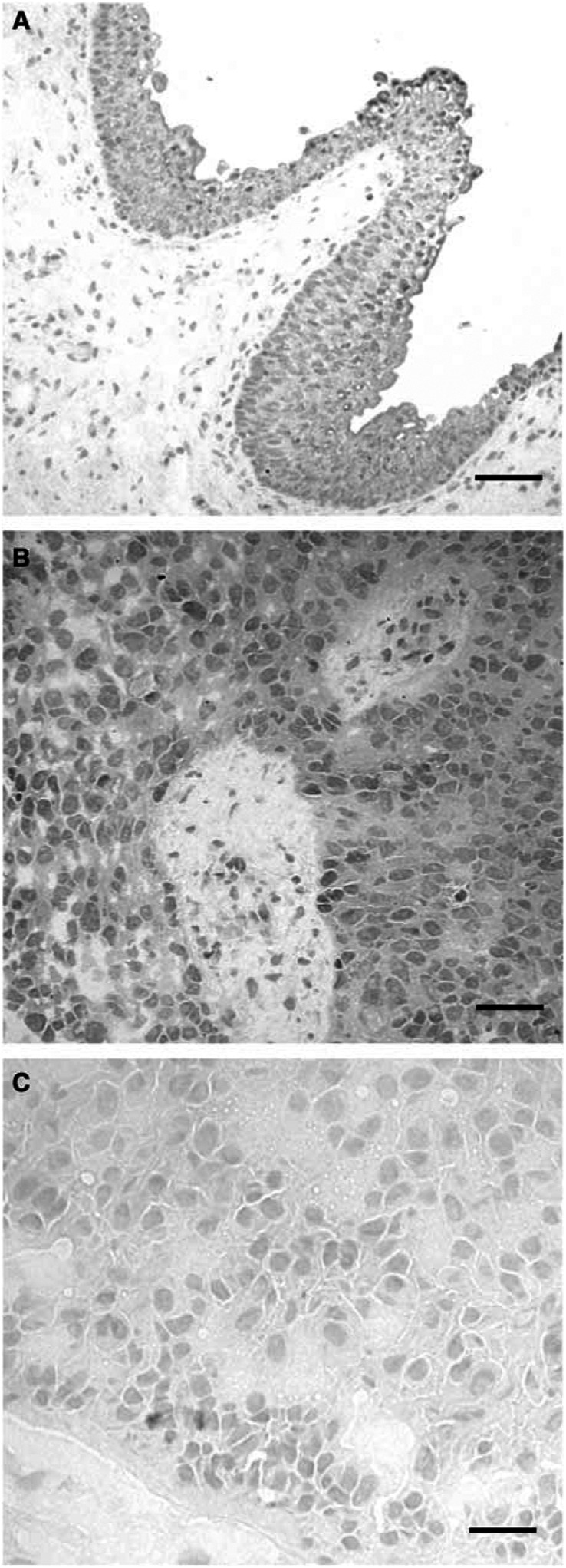
Immunohistochemical staining of Fas. (**A**) Normal urothelium shows staining of membranes and cytoplasm. (**B**) Grade 2 bladder cancer shows a homogeneous staining pattern. (**C**) Grade 3 bladder cancer is negative. Scale bar is 50 *μ*m long.

**Figure 5:**
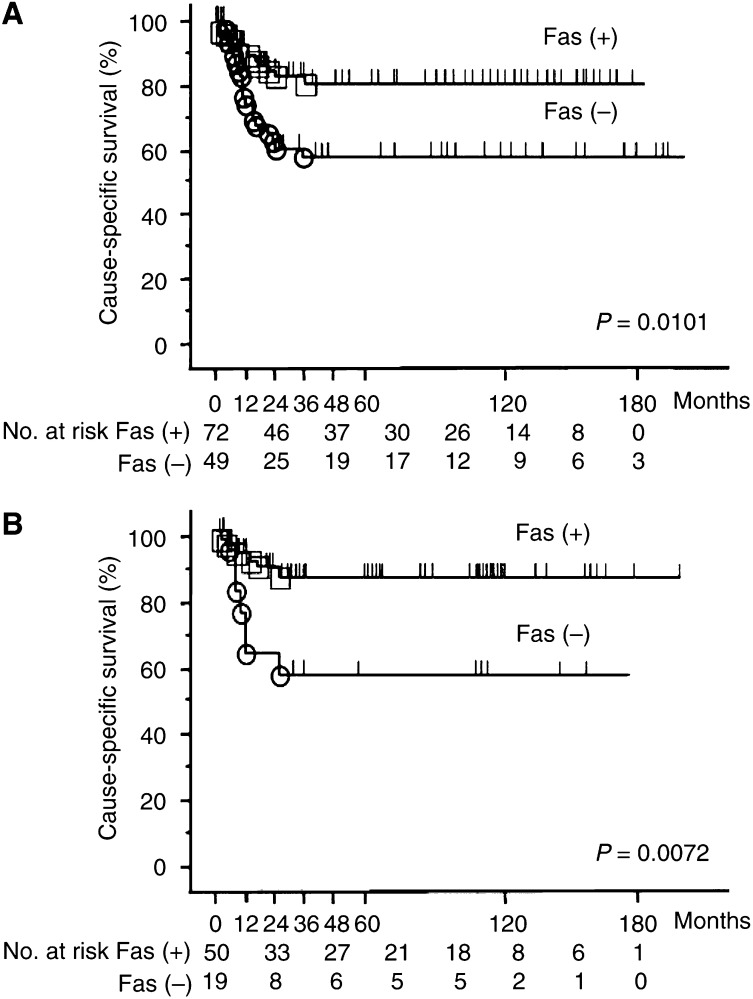
Desease-specific survival of UC patients according to Fas expression by the Kaplan–Meier estimation. (**A**) In all cases. (**B**) In grade 2 tumour cases only; Fas-negative status of tumours was associated with significantly poorer prognosis.

We demonstrated Fas expression in the normal urothelium and UC by immunohistochemistry. Fas was detectable in both the cytoplasm and plasma membrane of normal ulothelial cells and UC cells but not in the nuclei (Figure 4A and B). The normal urothelium showed a homogeneous expression in 27 out of 30 cases (90.0%), and the UC showed immunoreactivity for Fas in 72 of 123 cases (58.5%) (Table 3A). Fas expression inversely correlated with higher histopathological grade (G1: eight out of 11 (72.7%), G2: 50 out of 69 (72.5%), G3: 14 out of 43 (32.6%)). G1-2 tumours expressed Fas more frequently than G3 tumours (*P*<0.0001). A less frequent Fas expression was associated with a more advanced pathological stage (Ta: 13 out of 16 (81.3%), T1: 32 out of 52 (61.5%), T2: 10 out of 18(55.6%), T3: 12 out of 28 (42.9%), T4: three out of eight (37.5%)) (Table 3A). A significantly high proportion of Ta-1 tumours than T2-4 tumours expressed Fas (*P*=0.023). Fas-positive cases showed a significantly better prognosis (*P*=0.010, Figure 5A). When patients' survival was analysed in grade 2 tumours only, the same result was obtained (*P*=0.007, Figure 5B).

### FasL and DcR3 expression in normal urothelium and UC specimens

**Figure 6:**
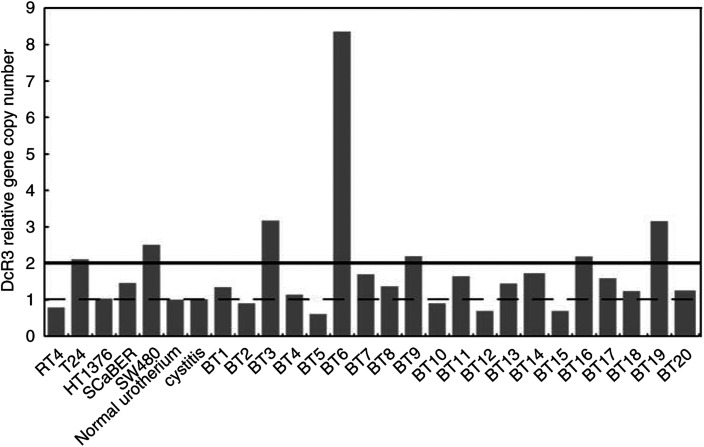
Quantitative PCR of DcR3. Relative gene copy number as a measure of DcR3 gene amplification in human bladder cancer cell lines and specimens. The colon cancer cell line SW480 was used as a positive control. As an internal control to confirm the amount of genomic DNA in the template, real-time PCR for *β*-actin was performed.

Decoy receptor 3 for FasL gene amplification was examined in cell lines and 20 bladder cancer specimens by real-time quantitive PCR. The T24 cell line and five of 20 UC had a copy number of two or more (Figure 6). We analysed the characteristics of five cases of DcR3 gene amplification. DNA amplification was not directly associated with the immunohistochemical pattern of DcR3 staining and clinical parameters in these patients. Fas ligand and DcR3 were also expressed in both the cytoplasm and plasma membrane. The expression of FasL was detected in UC (37.6%) and normal urothelium (23.3%, Table 3B). Fas ligand expression was present more frequently in G1-2 than in G3 tumours (*P*<0.0001), and tended to be more frequent in Ta-1 than T2-4 (*P*=0.061). DcR3 expression was detected in UC (90.0%) and normal urothelium (80.7%). The expression of DcR3 correlated with neither histopathological grade nor pathological stage (Table 3B). The expression of these two molecules did not have an association with disease-specific survival.

## DISCUSSION

Previously, we have demonstrated the inverse correlation of Bcl-2 protein level with sensitivity to ADM treatment in TCC cell lines (Bilim *et al*, 1997). Besides, ADM treatment alone did not have any direct influence on the expression level of antiapoptotic Bcl-2 or Bcl-xL. Here, we have got evidence that Bid, which is a proapoptotic member of Bcl-2 family proteins, was processed to its cleaved active form in a dose-dependent manner in TCC cells including T24, a multidrug-resistant cell line (Figure 2C). Moreover, ADM triggered procaspase-8 cleavage into active subunits (Figure 2C) and drastic increase of IETD-caspase-8-specific activity in susceptible cells (data not shown). These events suggested that ADM treatment could activate Fas and subsequent apoptotic signal transduction in TCC. To confirm or disprove this working hypothesis and get direct evidences, we proceeded to functional *in vitro* assays.

The expression of Fas was observed in each of four UC cell lines of different malignant potential and morphological type. Expression levels were similar among them. Despite the expression of Fas, treatment with CH-11 Fas-activating antibody alone did not have any cytotoxic effect in either T24 or RT4. However, we obtained a synergistic effect with ADM and CH-11 both in T24 and RT4. Furthermore, this synergic effect was demonstrated with low-dose CH-11. On this occasion, we did not find any upregulation of Fas expression after the exposure to ADM, and ZB4 Fas-blocking antibody did not affect the viability of either naïve or ADM-treated cells. These findings suggested that the apotosis induction by CH-11 is not dependent on the quantity of Fas receptor, and the main regulation mechanism might exist somewhere downstream in the signal cascade.

Published studies have shown that apoptosis through the activation of several caspases by chemotherapy using ADM did not depend on Fas–FasL interaction in other cancers (Gamen *et al*, 1997; Landowski *et al*, 1999), among them ADM downregulated FLIP, an antiapoptotic downstream molecule of Fas, in prostate cancer cell lines, which lead to sensitise cancer cells to death receptor-mediated apoptosis (Kelly *et al*, 2002). However, we did not find any correlation of FLIP expression with ADM susceptibility in UC cell lines (data not shown). We had reported that ADM triggered a type 2 apoptosis cascade, mitochondrial pathway, in UC (Bilim *et al*, 1997). On the other hand, several investigators showed that ADM treatment induced Fas or FasL upregulation and led to apoptosis via Fas–FasL interaction (Friesen *et al*, 1996; Mizutani *et al*, 1997; Villunger *et al*, 1997). The present study was not consistent with these previous reports; ADM treatment sensitised TCC cells to CH-11 without change in Fas expression level. This synergistic effect can be explained by the amplification of the downstream apoptosis cascade through the convergence of type 1 and 2 cascades activated by Fas and ADM, respectively. The convergent point might be in mitochondria, and the regulator of signal downstream could be proapoptotic Bcl-2 family proteins, such as Bid and Bax. There is increasing evidences from other tumour cites showing the role of Bid or Bax as regulator of apoptosis (Fukazawa *et al*, 2003; Schniewind *et al*, 2004). Direct induction of Fas-mediated apoptosis may be a promising strategy for an anticancer therapy particularly in combination with chemotherapy. However, severe systemic and potentially lethal toxicity by the attempts to administrate Fas agonists has been the major obstacle for its use of anticancer therapy. To avoid these harmful adverse events, intraperitoneal injection of Fas was tried and it revealed antitumour effect without systemic toxicity (Rensing-Ehl *et al*, 1995). In addition, presence of glycosaminoglycans covering the normal urothelium might be a natural barrier to prevent adverse effects of Fas agonist to the Fas presenting normal urothelial cells (Parsons *et al*, 1990; Shimizu *et al*, 2001). Thus, less toxic and more efficient antitumour effects may be expected by intravesical administration of the reagents, whereas further examination is warranted in addition to our data presented here.

We investigated the expression of Fas, FasL and DcR3 in 123 UC surgical specimens as well as 30 normal urothelial specimens by immunohistochemistry. It was found that loss of Fas was associated with clinical parameters. The majority of normal urothelial specimens expressed Fas, whereas in UC, Fas was downregulated. Furthermore, Fas immunoreactivity inversely correlated with higher pathological grade (*P*<0.0001), higher tumour stage (*P*=0.023) and shorter cause-specific survival (*P*=0.010). The disappearance of Fas may be a result of cancer progression, manifesting most apparently with dedifferentiation of cancer cells. In the present study, different tumours of the same grade, the most common criteria of cell malignancy and differentiation status, revealed various Fas status, which correlated with the prognosis. Therefore, we assumed that Fas on UC cells might have its function as a death receptor, and decreased Fas may contribute to acquisition of higher malignant potential. These results conflicted with two previous reports in which Fas expression had no correlation with clinicopathological variables (Lee *et al*, 1999; Giannopoulou *et al*, 2002). Recently, Maas *et al* (2004) reported that Fas downregulation was related to higher tumour stage the same as ours. On the contrary, they did not observe correlation with tumour grade and survival. In these studies, different antibodies were used for IHC (rabbit polyclonal C-20 or N-18, Santa Cruz); moreover, the results were based on a small number of patients. Fas downregulation is recognised as one of the mechanisms for evading the Fas–FasL system in other types of tumours, and a similar loss of Fas was shown to be a negative prognostic factor in acute lymphoblast leukaemia (Suminoe *et al*, 2004), lung cancer (Koomagi and Volm, 1999) and pancreatic cancer (Bernstorff *et al*, 2002). In the present study, grade 2 UC were presumably heterogeneous in terms of biological characteristics. Namely, some grade 2 UC progressed dramatically (*n*=13) while others showed no recurrence (*n*=56). Therefore, new factors, which can be used to subclassify grade 2 tumours, are needed for determining treatment strategy in this category. The current study suggests that grade 2 UC could be subclassified according to Fas expression. As was observed here, Fas-negative patients had a poor prognosis, which was also true of grade 2 tumours, indicating the possibility of applying Fas expression as a useful prognostic parameter in grade 2 UC.

Another aspect of the Fas–FasL system is FasL expression in cancer cells. Recently, Chopin *et al* (2003) reported FasL expression in a series of 44 UC specimens and showed that UC acquired a functional FasL with progression to a higher grade and stage. They applied A11 and H11 (Alexis, Lausen, CH) clones of FasL antibodies for immunohistochemical staining in this rather small patient cohort. Our results also demonstrated that FasL was expressed more frequently in UC than normal urothelium. However, FasL expression showed an inverse correlation with tumour grade and stage. This discrepancy cannot be fully explained, but it may be due to the use of different clones of antibodies and the large gap in patient number. There is problem in the reliability of antibodies for FasL, and several papers have tested their specificity; many clones including A11 and H11were listed as unreliable, on the other hand, because clone Q-20 we used had not been examined, we cannot give any other evidences besides our own results for the reliability of the antibody, but other clones of Santa Cruz were considered not applicable for IHC (Strater *et al*, 2001). As for the patients' survival, FasL expression showed no correlation with it. Our results on FasL do not support the Fas–FasL tumour counterattack theory in UC. In addition, DcR3, a decoy receptor for FasL, drew attention as a new molecule working to evade activation of the Fas–FasL system (Pitti *et al*, 1998). In several other malignancies, DcR3 gene amplification and expression were reported (Roth *et al*, 2001; Mild *et al*, 2002). This study is the first report on the gene and protein status of DcR3 in normal urothelium and UC. Whereas amplification and expression were seen in some cases of UC, DcR3 showed no correlation with tumour grade, tumour stage or patients' survival.

In conclusion, among Fas-related molecules in UC, Fas and the absence thereof may have the greatest impact on tumour progression through evading apoptosis leading to a poorer prognosis. Thus, the loss of Fas was suggested to be a crucial factor for selecting patients requiring more aggressive treatment. This is especially important for G2 tumours. Furthermore, low-dose anti-Fas-activating mAb sensitised resistant cells to ADM, and this synergistic effect was independent from expression level of Fas receptor, although the presence of Fas was essential. This effect could be applied in the development of new treatment strategy for UC patients with multidrug-resistant tumours.

## Figures and Tables

**Table 1:** Patient characteristics

Mean age (range) years	66.9 (41–89)
Male/female	92/32
Renal pelvic cancer	18
Ureteral cancer	20
Bladder cancer	86
(TUR-BT/total cystectomy)	(37/49)
Histopathological grading	
G1	11
G2	69
G3	43
Pathological staging	
Ta	17
T1	52
T2	18
T3	28
T4	8
Mean follow up (range), months	64.3 (0–192)

TUR-BT: transurethral resection of bladder tumour.

**Table 2:** Primers sequences for RT–PCR and real-time PCR

**Specificity forward (5′ → 3′)**	**Reverse (5′ → 3′)**	**Product size (bps)**	**Annealing T. (°C)**
RT–PCR			
Fas atgctgggcatctggaccct	gccatgtccttcatcacacaa	335	58
FasL aactcaaggtccatgcctctg	ggtgagttgaggagctacagaca	553	58
DcR3 gccactacacgcagttctggaacta	gaggaagagcctggcacattgaggg	336	60
DR4 attgtacgccctggagtgac	aaggacacggcagagcctgtgccat	741	60
DR5 ctgaaaggcatctgctcaggtg	cagagtctgcattaccttctag	582	58
DcR1 gccggaagtgtagcaggtg	ggggcaggggcaggcgtttct	546	58
DcR2 cttttccggcggcgttcatgtccttc	gtttcttccaggctgcttccctttgtag	463	58
β-ACT aatgcttctaggcggact	actcccagggagaccaaa	533	58
Real-time PCR			
DcR3 cttcttcgcgcacgctc	atcacgccggcaccag		

**Table 3A:**
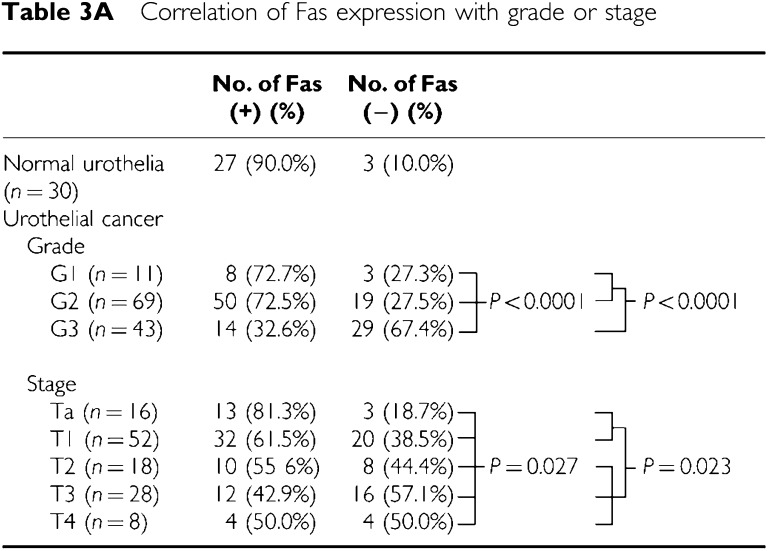
Correlation of Fas expression with grade or stage

**Table 3B:**
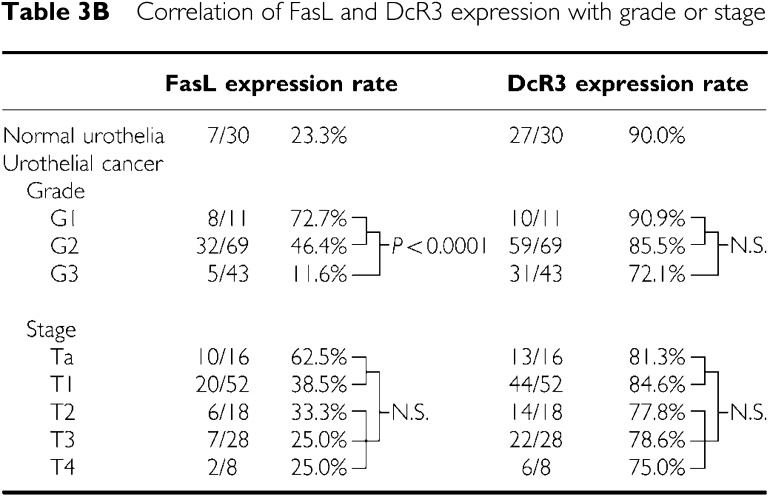
Correlation of FasL and DcR3 expression with grade or stage
